# Variant to Gene Mapping to Discover New Targets for Immune Tolerance

**DOI:** 10.3389/fimmu.2021.633219

**Published:** 2021-04-15

**Authors:** Parul Mehra, Andrew D. Wells

**Affiliations:** ^1^ Department of Pathology, The Children’s Hospital of Philadelphia, Philadelphia, PA, United States; ^2^ Department of Pathology and Laboratory Medicine, Perelman School of Medicine, University of Pennsylvania, Philadelphia, PA, United States

**Keywords:** genome-wide association studies, single nucleotide polymorphism, autoimmunity, multi-omics, immune tolerance, variant-to-gene mapping

## Abstract

The breakdown of immunological tolerance leads to autoimmune disease, and the mechanisms that maintain self-tolerance, especially in humans, are not fully understood. Genome-wide association studies (GWAS) have identified hundreds of human genetic loci statistically linked to autoimmune disease risk, and epigenetic modifications of DNA and chromatin at these loci have been associated with autoimmune disease risk. Because the vast majority of these signals are located far from genes, identifying causal variants, and their functional consequences on the correct effector genes, has been challenging. These limitations have hampered the translation of GWAS findings into novel drug targets and clinical interventions, but recent advances in understanding the spatial organization of the genome in the nucleus have offered mechanistic insights into gene regulation and answers to questions left open by GWAS. Here we discuss the potential for ‘variant-to-gene mapping’ approaches that integrate GWAS with 3D functional genomic data to identify human genes involved in the maintenance of tolerance.

## Introduction

Immune tolerance is established through clonal deletion during development of the immune repertoire, and is reinforced in the periphery through cell-intrinsic and -extrinsic mechanisms that limit activation and differentiation. Breakdown of central or peripheral tolerance can lead to autoimmunity (1), thus understanding the molecular and cellular mechanisms that control tolerance promises opportunities to therapeutically reprogram the immune system to treat inflammatory disease. The study of rare spontaneous or engineered monogenic mutations in the mouse have contributed significantly to our understanding of tolerance and autoimmunity. However, autoimmune disease in humans is relatively common, and the genetics of predisposition is complex, polygenic, and heavily influenced by environmental factors. Hundreds of genetic loci influencing susceptibility to various autoimmune and inflammatory disorders have been discovered by genome-wide association studies (GWAS) in humans, and in many cases have confirmed mouse models and yielded novel insights into mechanisms underpinning disease (2). Despite this, the identity of causal variants and their target genes remain largely unknown because variants rarely alter protein coding sequences. Instead, approximately 90% of immune disease-associated variants are located in non-coding regions, and integration of GWAS with *cis*-regulatory element annotations in immune cell types has shown that ~60% map to immune cell enhancers (3–8). Identification of causal variants and their target genes are the next, necessary steps for understanding the molecular mechanisms by which genetic variation regulates immune tolerance, and for identifying new drug targets for treating autoimmune disease. This perspective discusses cellular and molecular mechanisms involved in the breakdown of immunological tolerance, as well as the potential of functional genomic approaches to define target genes in specific immune cell types to better understand the mechanism of autoimmunity.

## Maintenance of Immunologic Tolerance in Humans And its Role in Autoimmunity

Central tolerance takes place in the thymus and bone marrow through apoptotic deletion of autoreactive lymphocytes. Without negative selection, T and B cells respond to self-antigens and attack self-tissues, resulting in autoimmune pathologies (9–12). Autoreactive lymphocytes that escape negative selection in the primary lymphoid organs are normally held in check by additional, peripheral tolerance mechanisms that operate to dampen activation in secondary lymphoid tissues. The discovery of regulatory T cells (Tregs) represents a fundamental advance in our understanding the cellular basis of immune tolerance in the context of autoimmunity, transplantation, and cancer (13). The monogenic immune disorder IPEX (Immunodysregulation, Polyendocrinopathy, Entereopathy, X-linked syndrome) provides an example of a breach in tolerance in which mutations in the forkhead box P3 (*FOXP3*) gene leads to loss of Treg and/or their function (14, 15). A recent study used the combination of deep flow cytometric and single-cell RNA-seq profiling of Tregs and conventional CD4+ T cells from IPEX patients to identify gene signatures associated with IPEX (16). Using CRISPR-Cas9 genome editing, Goodwin et al. modified the FOXP3 gene in human hematopoietic stem cells to enhance the stability of FOXP3 expression and the suppressive capacity in Tregs (17). A similar gene editing approach in a mouse model resulted in the generation of “super Tregs” capable of resolving the severe inflammation in IPEX-like disorders by modulating the chromatin modifier Brg1 (18). Studies of monogenic disorders like IPEX could lead to new biomarkers and therapeutic strategies for managing polygenic autoimmune disorders. Dysregulation of the IL-1B, inflammasome-related proteins (NLR genes), and type-I interferon pathway represent additional mechanisms known to contribute to the loss of self-tolerance (19, 20), and need to be further studied to understand the role of dysregulated tolerance in human autoimmunity disease.

In contrast to monogenic diseases, autoimmune disorders such as systemic lupus erythematosus (SLE), rheumatoid arthritis (RA), multiple sclerosis (MS), and type 1 diabetes (T1D) are heritable diseases involving more than one gene and cell type in their etiology. Immune dysregulation observed in patients include enhanced activation of autoreactive Th1 and Th17 CD4 cells (21, 22), CD8 suppressor T lymphocytes targeting self-antigens in the CNS (23, 24), defective regulatory T cells (25–27), autoreactive B cells (28, 29), and aberrant T lymphocyte signaling and cytokine production (30–34). Given the complexity and heterogeneity associated with polygenic autoimmune disease, there is a need for better therapeutic approaches that specifically target pathogenic mechanisms. Understanding the specific cell types and functions dysregulated in autoimmune disease offers the potential for new drug targets and therapeutic approaches (35, 36). Tregs have been shown to be defective in the autoimmune disease settings, and ex vivo-expanded Tregs isolated from T1D patients have been used in phase I clinical trial as adoptive immunotherapy in T1D. In this trial expanded cells were found to retain their phenotype, TCR diversity, and functional capacity in patients for long periods (37). A transcriptomic study conducted in SLE patients showed that gene signatures associated with interferon signaling is significantly dysregulated (38), although current efforts targeting IFN in SLE have not been successful. A more recent study in this field profiled six major immune types in SLE patients by single-cell RNAseq and found a unique set of genes in monocytes, including two well-known immune modulators for SLE and RA therapeutics (*TNFSF13B/BAFF*: belimumab and *IL1RN*: anakinra, respectively) (39). A single cell transcriptomic study in Crohn’s patients revealed a gene program associated with inflamed tissue, consisting of genes expressed by plasma cells, inflammatory mononuclear phagocytes, and activated T cells (40). These cutting-edge approaches are changing our appreciation of the complexity and heterogeneity of autoimmune disorders, and are helping to discover new therapeutic strategies and identifying new therapeutic biomarkers.

## Complex Genetics and Epigenetics of The Loss of Immunologic Tolerance in Humans

Genetic predisposition and epigenetic modifications are implicated in the loss of tolerance and autoimmunity, and emerging genomic technologies are enabling comprehensive interrogation of genetic variants that contribute to autoimmune disease risk. Genome-wide association studies have implicated hundreds of loci in disease susceptibility, many of which are disease-specific. However, a number of risk loci are shared across multiple diseases, suggesting the involvement of common pathways associated with the loss of tolerance. The MHC locus is genetically associated with all autoimmune diseases (41, 42). Much of this linkage is thought to be driven by HLA coding polymorphisms that affect self-peptide binding, however, this region contains over 200 genes, and high polymorphism and linkage disequilibrium across the locus presents technical challenges for identifying risk alleles and alternative causal genes. *CTLA4* has been linked with T1D (43) and auto-antibody positive RA (44). This immunoglobulin superfamily member is expressed on the surface of conventional and regulatory T cells that transmits an inhibitory signal for T cell activation and strips costimulatory ligands from antigen presenting cells. A non-synonymous variant in *PTPN22* was shown to be associated with many autoimmune diseases, including T1DM, RA, SLE and Graves disease (45–47). The risk variant in *PTPN22* gene affects the binding of lymphoid phosphatase (LYP) to the signaling suppressor SRC kinase and ultimately affects the signaling pathways during T cell and B cell receptor response.

Several approaches have been used to map autoimmune disease variants to effector genes in recent years. The advent of the Illumina Infinium SNP Immunochip has helped to fine map many autoimmune GWAS loci including SLE. In a study of lupus, researchers used a random forest machine learning method to integrate Immunochip genotyping and T and B cell RNA-seq analysis from SLE patients and healthy control subjects, identifying three novel genes (*ZNF804A*, *CDK1*, and *MANF)* that were not previously been associated with SLE or any other autoimmune disorder (48). To functionally validate the allele specific expression pattern of 3,000 genes identified by genotype data from the Immunochip, an eQTL analysis was performed in B and T cells from healthy donors which leads to the involvement of *cis*-regulatory SNPs in gene regulation. Conclusively, six SLE associated genes found to be regulated by *cis*-rSNPs were *IKZF1*, *NCF2*, *IL12A* and *TNIP1* in B cells and *ANK3*, and *PHRF1* in T cells (48). The combination of machine learning and allele-specific transcriptome analysis represents a valuable tool for validation of target genes associated with disease risk and offers a functional follow-up strategy to test these molecular targets under clinical settings.

A growing body of work links epigenetic modifications in immune cells with autoimmune disease risk. Epigenetic processes like DNA methylation and histone modification contribute to the expression of genes associated with disease (49, 50), and characterization of epigenetic factors could provide deeper insight into the onset and progression of disease. An example is the association of SLE with perturbed DNA methylation, a process that influences expression of cytokines like IL-2, IFN-gamma, IL-4 and IL-13 (51–53), and the Treg transcription factor FOXP3 (54). Naïve T cells from SLE patients exhibit global hypomethylation due to decreased DNMT1 activity (55), with specific genes such as CD11a (*ITGAL*), perforin (*PRF1*), CD70 (*TNFSF7*), and CD40LG (*TNFSF5*) showing decreased DNA methylation (56). In addition, altered DNA methylation patterns at STAT3, IL6 and CXCL12 has been associated with RA pathogenesis (57), and expression of the epigenetic enzymes DNMT1, MBD3 and MBD4 were found to be decreased in systemic sclerosis patients. The latter was associated with increased expression of *CD40L*, *CD11a*, and *CD70* (58). As these epigenetic modifications alter gene expression programs of immune cells, epigenetics-based drugs and editing tools are emerging as a promising therapeutic option to restore healthy epigenetic landscapes under disease settings.

Integration of transcriptomic and epigenomic data with GWAS data provides a genome-scale view of the potential function of autoimmune risk variants in disease relevant cell-types. The Encyclopedia of DNA Elements (ENCODE) database has been used to corelate known genetic variants with histone H3 lysine 4 tri-methylation (H3K4me3) marks to identify cell types associated with particular autoimmune disease. Examples are studies that colocalized 31 RA-associated SNPs with H3K4me3 marks in CD4+ T cells (59), and colocalization of RNA-seq and ChIP-seq signals for the H3K4me1 and K3K27Ac enhancer marks in neutrophils and CD4+ T cells with JIA-associated variants in patients (60). Yuen et al. used publicly available ENCODE and Roadmap Epigenomics data generated in CD4+ T cells and B cells along with the ChIP-seq data generated in human neutrophils to examine the “epigenetic landscape” of SLE SNPs in a cell type-specific manner in adult immune cells (61). To identify whether immune disease variants regulate activation and differentiation, researchers profiled chromatin accessibility by ATAC-seq along with active enhancers and promoters by ChIPmentation-seq analysis in naïve and memory CD4+ T cells and macrophages. Using a newly developed statistical SNP enrichment method (CHEERS), the authors provided a comprehensive view of epigenetic mark enrichment at immune disease variants under specific activation and polarization conditions (62). The advent of single-cell genomics and gene editing technologies like CRISPR will allow functional validation of regulatory variants that influence immune tolerance and localize their effects to specific immune cell subsets.

## Statistical Linkage of Autoimmune Risk Variants With Tolerance Gene Expression

GWAS identifies large blocks (10–1,000 kb) of the genome that contain hundreds or thousands of SNPs, any of which could be causal (63), and colocalization studies like those described above have helped to identify potentially regulatory SNPs at GWAS loci. However, the vast majority of disease-associated SNPs and their associated epigenomic features are not located in gene promoters, and therefore the genes they regulate are not known. *cis*-eQTL (expression quantitative trait loci) analyses have been used to statistically link gene expression with SNP genotype. Most large eQTL studies so far have used peripheral blood expression data (64–67), and have linked ~42% of autoimmune sentinel SNPs to expression of a gene at the locus. A number of studies have shown that causal SNPs (e.g., in celiac disease and rheumatoid arthritis) disrupt transcription factor binding sites (68–70) and massively parallel reporter assays have identified SNPs that affect the activity of regulatory elements  (69). However, the majority of autoimmune loci lack eQTL support, likely due to use of data from undifferentiated and/or non-activated immune cells, as colocalization studies show that risk SNPs are enriched for functional marks mainly in stimulated and differentiated cell types (3). Using RNA-seq data collected from PBMC of 629 healthy patients, Ricano-Ponce et al. performed conditional *cis*-eQTL mapping and implicated 233 causal genes (e.g., *IL6, CXCR4, BCL-XL, MYC*), including 53 long non-coding RNAs, from 120 loci associated with 14 autoimmune disorders (64). Another study linked 39 lupus-associated variants to genes through the integration of GWAS and eQTL data from TwinsUK microarray and exon-level RNA-seq cohort in lymphoblastoid cell lines. This study identified novel, SLE-associated splice variants and novel candidate SLE eGenes, including SOCS1, CSK, and the transcription factor IKZF2 involved in Treg stabilization during inflammatory responses (71, 72). Importantly, these studies showed that more than half of the associated genes were not those nearest to the candidate SNP.

## Biophysical Linkage of Autoimmune Variants to Tolerance Genes Through Chromosome Conformation-Based Approaches

The human genome is organized in three-dimensional (3D) space in the nucleus into active and inactive compartments (73). Within active compartments, chromatin is organized into loops that can connect long-range regulatory elements with distant gene promoters. Recent high-throughput approaches for measuring the 3D structure of the genome in cells have provided new insights into global genome organization and the role of chromatin topology in genome function and dysfunction in health and disease. Two examples are studies by Jung et al. and Javierre et al. using a low-resolution Capture-HiC approaches to map the interactomes of ~18,000 human gene promoters in 27 human tissue/cell types (74), and ~30,000 promoter connectomes in 17 hematopoietic cell types (75). Genomic regions caught interacting with promoters were enriched for open chromatin, active histone marks, and disease-associated SNPs and eQTLs. Both studies were able to detect cell-type specific regulatory architectures, and the latter study also assembled a set of core genes connected to SNPs associated with multiple autoimmune disorders into an “autoimmune network” (75). Importantly, both studies found that less than 10% of disease-associated SNPs were connected to the nearest gene, further emphasizing that one cannot assume that risk SNPs (or other genomic features) regulate their nearest gene. Significant associations have been found between complex traits and gene deserts (>500 kb of genomic region which either lack protein coding sequence or annotated gene) which suggests that disease causing SNPs can affect gene expression by altering transcription factor binding to long-distance regulatory elements (76–78). Thus, integrating complexity of 3D genome into functional validation of GWAS studies can help uncover new insights in autoimmune disease pathogenesis.

In a more recent study, Su et al. used the combination of high-resolution promoter-Capture-C and ATAC-seq to map regulatory SLE variants to their target genes (79). Importantly, this study focused on follicular T helper cells (TFH) ‘caught in the act’ of helping B cells to produce antibodies in human tonsil. Unlike undifferentiated T cells or cell lines commonly used for these types of genome-scale studies, TFH play an active role in generating the pathogenic auto-antibodies characteristic of SLE, and represents a highly relevant target tissue for functional genomic analyses. This study identified ~400 accessible SLE variants connected to a network of 330 genes enriched for high expression in TFH and roles in T cell differentiation, humoral immunity, systemic autoimmune disorders, rheumatic disease, and type 1 diabetes. Remarkably, the physical SLE SNP-gene linkages measured in this one cell type confirmed one out of three linkages established statistically (eQTL) in a prior study (8). CRISPR/CAS9 genome editing validated novel SLE-associated regulatory elements that regulate TFH and SLE genes like *BCL6, CXCR5*, and *IKAROS*. A separate study combined the SNPs associated with 19 autoimmune diseases with cell-specific multi-omics approaches to develop an epigenetic weighted scoring method to evaluate the functionality of all noncoding autoimmune SNPs. The analysis also suggested long-range chromatin interactions between functional SNPs and distal target genes, highlighting the unique regulatory roles of noncoding SNPs associated with autoimmune diseases (80).

**Figure 1:**
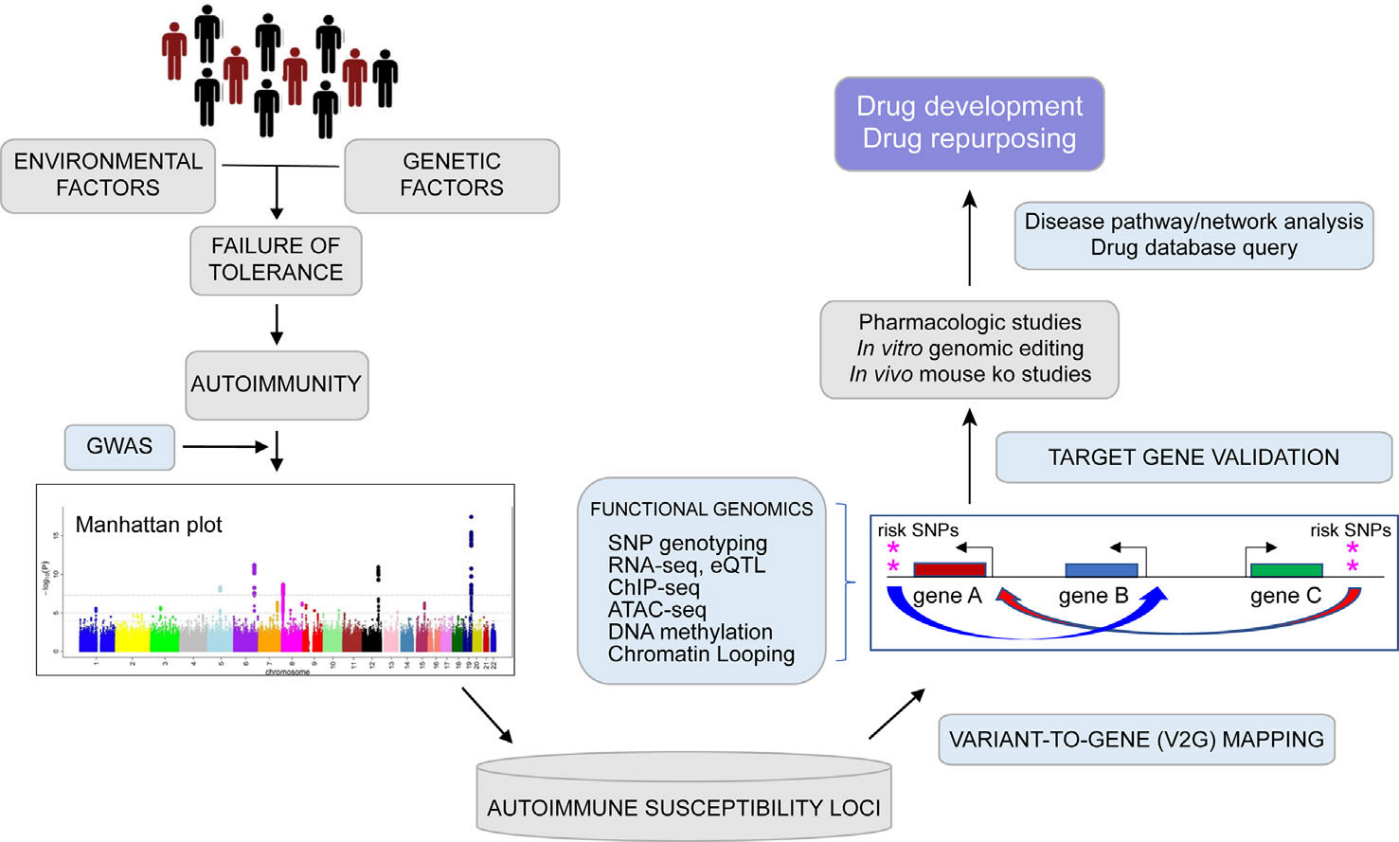
From genome to function: Graphical depiction of a pipeline leveraging genetic and epigenetic datasets to connect auto-immune disease associated variants to their target genes with focus toward drug development and repurposing. Genome wide association studies (GWAS) can identify multiple common genetic variants that confer risk for various diseases (as shown by Manhattan plot) including auto-immune disorders, but which variants are causal and which genes are involved remains largely unknown. Expression quantitative trait locus (eQTL) studies, high-resolution analysis of epigenomic and spatial organization can connect potentially functional SNPs with expression of putative disease genes in relevant cell types. Disease pathway exploration and experimental validation may lead to drug development and repurposing efforts.

In addition to revealing the disease-associated regulatory architectures of known autoimmune genes, spatial maps of variant accessibility and gene connectivity in immune cell types can be used to identify novel genes involved in tolerance and autoimmunity. For example, the study by Su et al. highlighted a set of genes identified by virtue of their physical connection to SLE variants that had no prior known role in lupus or TFH function. Two of these genes encode the kinases HIPK1 and MINK1, and genetic or pharmacologic inhibition of their function in human TFH cells resulted in reduced secretion of IL-21, a signature TFH cytokine required for class-switched antibody production by B cells. Targeting of HIPK1 in TFH reduced expression of the SLE genes *PTPN22, IL6R, IL2R, BACH2*, and *PD1* (79). The coalescence of state-of-the-art, genome-scale 3D-omic data from relevant immune cell types holds promise to further our basic understanding of the mechanisms that control immune tolerance, and point to novel targets for therapeutic intervention to treat and/or prevent autoimmune and inflammatory disorders (**Figure 1**).

## Conclusion

Detailed characterization of the functional effects of autoimmune disease-associated genetic variation on gene expression and immune cell function is of paramount significance to our understanding of immune tolerance. Interpreting SNP-trait associations requires integration of functional information from resources and repositories such as Genotype-Tissue Expression (GTEx), ENCODE, the Epigenomics Roadmap Project, and focused variant-to-gene (V2G) mapping studies like those described here. To overcome challenges like co-regulation of multiple genes and tissue heterogeneity, techniques must be fine-tuned to identify the most specific drug targets and biomarkers. Single-cell transcriptomic (scRNA-seq) and epigenomic (scATAC-seq) approaches offer the potential to dissect the contributions of cell, SNP, gene, and functional heterogeneity to immune disease. Tools for detecting and analyzing global genetic and epigenetic diversity are continuously evolving, and are on track to revolutionize our understanding of normal immune development and function, immune dysregulation and the breakdown of tolerance, and targets for new therapeutics to treat inflammation.

## Author Contributions

PM and AW conceptualized and wrote the manuscript. All authors contributed to the article and approved the submitted version.

## Funding

AW is funded by NIH grants DK122586, AI123539, AI130115, and AI054643, and by the Center for Spatial and Functional Genomics at the Children’s Hospital of Philadelphia.

## Conflict of Interest

The authors declare that the research was conducted in the absence of any commercial or financial relationships that could be construed as a potential conflict of interest.
